# Nasopharyngeal colonisation dynamics of bacterial pathogens in patients with fever in rural Burkina Faso: an observational study

**DOI:** 10.1186/s12879-021-06996-7

**Published:** 2022-01-04

**Authors:** Liesbeth Martens, Bérenger Kaboré, Annelies Post, Christa E. van der Gaast-de Jongh, Jeroen D. Langereis, Halidou Tinto, Jan Jacobs, André J. van der Ven, Quirijn de Mast, Marien I. de Jonge

**Affiliations:** 1 Department of Laboratory Medicine, Laboratory of Medical Immunology, Radboud university medical center, Nijmegen, the Netherlands; 2 Department of Medical Microbiology, Radboud university medical center, Nijmegen, the Netherlands; 3 Radboudumc Center for Infectious Diseases, Radboud university medical center, Nijmegen, the Netherlands; 4 Department of Internal Medicine, Radboud university medical center, Nijmegen, the Netherlands; 5 Institut de Recherche en Sciences de la Santé/Clinical Research Unit of Nanoro, Nanoro, Burkina Faso; 6 Department of Clinical Sciences, Institute of Tropical Medicine, Antwerp, Belgium; 7 Department of Microbiology, Immunology and Transplantation, KU Leuven, Leuven, Belgium

**Keywords:** Nasopharyngeal carriage, *Streptococcus pneumoniae*, *Haemophilus influenzae*, *Moraxella catarrhalis*, *Staphylococcus aureus*, *Klebsiella pneumoniae*, Burkina Faso

## Abstract

**Background:**

Nasopharyngeal colonisation with clinically relevant bacterial pathogens is a risk factor for severe infections, such as pneumonia and bacteraemia. In this study, we investigated the determinants of nasopharyngeal carriage in febrile patients in rural Burkina Faso.

**Methods:**

From March 2016 to June 2017, we recruited 924 paediatric and adult patients presenting with fever, hypothermia or suspicion of severe infection to the Centre Medical avec Antenne Chirurgicale Saint Camille de Nanoro, Burkina Faso. We recorded a broad range of clinical data, collected nasopharyngeal swabs and tested them for the presence of *Streptococcus pneumoniae*, *Haemophilus influenzae*, *Moraxella catarrhalis*, *Staphylococcus aureus* and *Klebsiella pneumoniae* by quantitative polymerase chain reaction. Using logistic regression, we investigated the determinants of carriage and aimed to find correlations with clinical outcome.

**Results:**

Nasopharyngeal colonisation with *S. pneumoniae*, *H. influenzae* and *M. catarrhalis* was highly prevalent and strongly dependent on age and season. Females were less likely to be colonised with *S. pneumoniae* (OR 0.71, p = 0.022, 95% CI 0.53–0.95) and *M. catarrhalis* (OR 0.73, p = 0.044, 95% CI 0.54–0.99) than males. Colonisation rates were highest in the age groups < 1 year and 1–2 years of age and declined with increasing age. Colonisation also declined towards the end of the rainy season and rose again during the beginning of the dry season. *K. pneumoniae* prevalence was low and not significantly correlated with age or season. For *S. pneumoniae* and *H. influenzae*, we found a positive association between nasopharyngeal carriage and clinical pneumonia [OR 1.75, p = 0.008, 95% CI 1.16–2.63 (*S. pneumoniae*) and OR 1.90, p = 0.004, 95% CI 1.23–2.92 (*H. influenzae*)]. *S. aureus* carriage was correlated with mortality (OR 4.01, p < 0.001, 95% CI 2.06–7.83), independent of bacteraemia caused by this bacterium.

**Conclusions:**

Age, sex and season are important determinants of nasopharyngeal colonisation with *S. pneumoniae*, *H. influenzae* and *M. catarrhalis* in patients with fever in Burkina Faso. *S. pneumoniae* and *H. influenzae* carriage is associated with clinical pneumonia and *S. aureus* carriage is associated with mortality in patients with fever. These findings may help to understand the dynamics of colonisation and the associated transmission of these pathogens. Furthermore, understanding the determinants of nasopharyngeal colonisation and the association with disease could potentially improve the diagnosis of febrile patients.

**Supplementary Information:**

The online version contains supplementary material available at 10.1186/s12879-021-06996-7.

## Background

The human nasopharynx is the main reservoir for well-known respiratory pathogens such as *Streptococcus pneumoniae*, *Haemophilus influenzae* and *Moraxella catarrhalis* [1–3]. It is also a secondary reservoir for *Staphylococcus aureus*, which preferentially colonises the nares but can be found throughout the respiratory tract as well as the gastrointestinal tract and the skin [4]. *Klebsiella pneumoniae*, a microorganism that primarily colonises the gastrointestinal tract, is occasionally found in the nasopharynx and is an important cause of community acquired pneumonia (CAP) in low-income countries [5–7].

Nasopharyngeal carriage of these bacteria is usually harmless and asymptomatic. However, under specific conditions they can enter the lungs, the bloodstream or even the central nervous system and cause systemic disease [1–5]. Additionally, the human nasopharynx acts as a hub from where these pathogens are transmitted to other people, leading to spread within the population.

Information on the epidemiology and dynamics of nasopharyngeal carriage of *S. pneumoniae*, *H. influenzae*, *M. catarrhalis*, *S. aureus* and *K. pneumoniae* is limited. The notable exception to this is the effect of pneumococcal vaccination on nasopharyngeal carriage of *S. pneumoniae* in children in West Africa [8–11]. Another known fact is that there are major regional differences in the rates of carriage [12]. A relatively high nasopharyngeal prevalence of *K. pneumoniae* has been reported in low-income countries, which could explain why this bacterium is a much more common cause of CAP in these countries, as compared to high-income countries [6, 7, 12]. Some research has also been published on the influence of season, age, sex and rurality, mainly on *S. pneumoniae* [8, 9, 13–17]. Data on nasopharyngeal colonisation with *H. influenzae*, *M. catarrhalis*, *S. aureus* and *K. pneumoniae* in West Africa are sparse, especially for adults and with regard for seasonal influences [8, 10, 16, 18–20].

Considering these gaps in knowledge, we aimed to perform a cross-sectional study with an adequate number of inclusions throughout the year so as to create a better understanding of the epidemiology of nasopharyngeal carriage of these pathogens. We believe these data could provide insight into the epidemiological determinants of the infections caused by the studied pathogens. This knowledge may also be a starting point for determining which interventions could lead to a lower incidence of these infections. We hypothesized that season was a major determinant of carriage for nasopharyngeal carriage of *S. pneumoniae*, *H. influenzae*, *M. catarrhalis*, *S. aureus* and *K. pneumoniae* and aimed to document the seasonal dynamics of carriage of these bacteria. We further hypothesized that there would be a correlation with HIV, malaria and/or tuberculosis and that carriage of one pathogen could influence carriage of the others. Lastly, we aimed to show the clinical relevance of our findings by demonstrating a correlation between carriage and clinical disease and/or outcome.

## Methods

### Study area and design

This study was designed as a side project to the PALUBAC study, which was set up to test and optimise an algorithm to distinguish malaria from bacterial infections in acutely ill febrile patients. Inclusion and exclusion criteria of the PALUBAC study and a complete report on which data were collected have been published previously [21–23]. Briefly, participants were enrolled between March 2016 and June 2017 at the Centre Médical avec Antenne Chirurgicale (CMA) Saint Camille de Nanoro, Burkina Faso. Nanoro is located in the central-western part of the country. It has a rainy season from June to October, with an average rainfall of 450–700 mm per year, and a dry season from November to May [24]. The Nanoro department is a rural area with an average population density of 139/km^2^. The majority (90.13%) of the population lives in rural conditions. The principal activity of the inhabitants is agriculture, mainly subsistence farming. 50.57% of inhabitants are children under 15 years of age [25]. The national immunisation program has included the conjugate *H. influenzae* type B vaccine since 2006 and the 13-valent conjugate *S. pneumoniae* vaccine since 2013. Children under 5 years of age receive seasonal malaria chemoprophylaxis from July to October. Participants were eligible for inclusion if they had a fever (temperature ≥ 38.0 °C), hypothermia (temperature ≤ 35.5 °C), a reported history of fever or hypothermia up to 48 h prior to presentation at the health facility or a suspicion of severe infection. Patients under 3 months of age and patients with fever or hypothermia lasting longer than 7 days were excluded. From March until November 2016, only hospitalised patients were enrolled. Thereafter, until the end of the study in June 2017, non-hospitalised patients were also enrolled because of insufficient study recruitment [23].

Basic demographic and clinical data were recorded on a standardized case report form by study nurses. Upon inclusion, a blood sample was taken, as well as a nasopharyngeal swab in UTM medium. The blood sample was used for malaria diagnostics (rapid diagnostic tests, thin and thick film microscopy), blood culture and basic hemocytometry. Cultures of urine, stool, pus or cerebrospinal fluid and imaging diagnostics (chest X-ray or abdominal echography) were performed only when indicated, as was serology for specific infectious diseases (e.g. HIV). Residual blood and the nasopharyngeal swabs were stored at − 20 °C and shipped to the Radboud university medical center in Nijmegen, the Netherlands. There, all blood samples were tested for malaria using quantitative polymerase chain reaction (qPCR). qPCR for *Salmonella* spp., *S. pneumoniae*, *H. influenzae* and *S. aureus* was performed on blood samples of patients with negative blood cultures. qPCR methods for the detection of bacterial pathogens in blood samples were described in a previous publication [23]. The detection limit was 75 copies of DNA per ml of blood.

For the purpose of data analysis, ‘bacteraemia’ was defined as the presence of a pathogenic bacterium either in blood culture or in blood qPCR. Upon discharge, a final diagnosis made by a local physician was recorded on the case report form. For children below 5 years of age, pneumonia was defined according to the WHO criteria [26]. For older children and adults, pneumonia was defined as patients having fever and one or more signs of dyspnoea (rapid breathing, nasal flaring, chest indrawing, saturation below 95%, subjective feeling of dyspnoea), combined with either an X-ray showing pulmonary infiltrate or chest auscultation suspect for pneumonia. The criteria for a diagnosis of tuberculosis were cough lasting ≥ 14 days and weight loss, in combination with (1) a positive *Mycobacterium tuberculosis* PCR (GeneXpert® MTB/RIF) on sputum, (2) acid-fast bacteria in Ziehl–Neelsen stain on sputum or (3) typical caseating granulomas on X-ray.

### qPCR analysis on nasopharyngeal swab samples

qPCR for *S. pneumoniae*, *H. influenzae*, *M. catarrhalis*, *S. aureus* and *K. pneumoniae* was performed on all nasopharyngeal swabs. Stored swab samples were thawed on ice and vortexed. From each sample, 200 µl was aliquoted into a 96-wells plate. The plate was incubated for 15 min at 93 °C to lyse the bacteria [27]. The qPCR was performed in five monoplex reactions, using the Bio-rad CFX96 Touch Real-Time PCR Detection System. All reactions were performed in a 10 µl final volume containing 1 µl bacterial lysate, 5 µl SsoAdvanced™ Universal Probes Supermix (Bio-Rad), 400 nM of each primer and 200 nM probe. For details on primers and probes, see Additional file 1: Sheet 1. Every 96-wells plate contained, in duplicate, a no template control and a seven step tenfold serial dilution of a positive control, starting at approximately 10 ng/µl. The qPCR program consisted of 3 min of incubation at 95 °C followed by 50 cycles of 10 s at 95 °C and 20 s at 60 °C for all targets except for *K. pneumoniae*, for which the cycles were 10 s at 95 °C and 20 s at 65 °C. Fluorescence was measured after each cycle. In order to accurately compare results within targets, the baseline threshold was adjusted so that the log 3 dilutions of the positive control had the same Cq value per target for all 12 plates. Cq cutoff was determined per target using data on negative controls and samples that were tested in duplicate. The detection limit of the qPCR was calculated to be below ten copies of DNA per µl UTM medium for all targets. All qPCR results can be viewed online (Additional file 2).

### Statistical analyses

Data were analysed using SPSS Statistics 25.0.0.1 (IBM, Armonk, New York, USA). Carriage prevalences were estimated from logistic regression models adjusted for by sex (male), age (5.0 years) and/or month of inclusion (January). Odds ratios and p-values were calculated after adjustment for age, sex and month of inclusion using logistic regression. p Values below 0.05 were considered statistically significant. The SPSS output file can be viewed online (Additional file 3).

### Ethical approval

The study protocol was approved by the national ethics committee of Burkina Faso, the institutional review board of IRSS, the ethical committee of the University of Antwerp and the internal review board of the Institute of Tropical Medicine Antwerp, Belgium. Written informed consent was obtained from all participants or their parents/legal guardians with additional assent from those aged 7 to 15 years.

## Results

### Study population and general colonisation rates

A total of 984 patients met the inclusion criteria for the PALUBAC study, 54 of whom were not included: 27 patients refused participation, 15 patients were referred to another hospital before they could be included, 4 patients died before they could be included and 8 were not included for other reasons. A total of 930 patients were enrolled, 6 of whom were excluded from this analysis because they did not provide a nasopharyngeal swab, resulting in a final number of 924 patients.

More patients were included in the dry season, as compared to the rainy season (83 vs 68 patients per month) and more males than females were included (533 males vs 391 females) (Table 1 and Additional file 1: Sheet 2). The age of included patients ranged from 3 months to 90 years, with a median of 5.0 years. Out of 565 children below the age of 12 years, 62 (11.0%) were classified as malnourished, which was defined as a WHO Anthro weight-for-height z-score < 2.

**Table 1:** General characteristics of the study population

Characteristic	n	%
*Sex*		
Female	391	42.3
Male	533	57.7
*Age group*		
< 1 years	152	16.5
1–2 years	213	23.1
3–5 years	115	12.4
6–11 years	85	9.2
12+ years	359	38.9
*Malnutrition*^§^		
< 1 years	24	15.8
1–2 years	32	15.0
3–5 years	6	5.2
6–11 years	0	0.0
*Malaria status*		
Microscopy positive	247	26.7
Microscopy negative, PCR positive	96	10.4

^§^Malnutrition was defined as a WHO Anthro weight-for-height z-score < 2. Data on malnutrition were only recorded for patients below 12 years of age

After admittance, 713 patients (77.2%) were eventually discharged because they had improved clinically, 128 (13.9%) were referred to another hospital, 50 patients (5.4%) died and 33 (3.6%) left against medical advice.

**Fig. 1:**
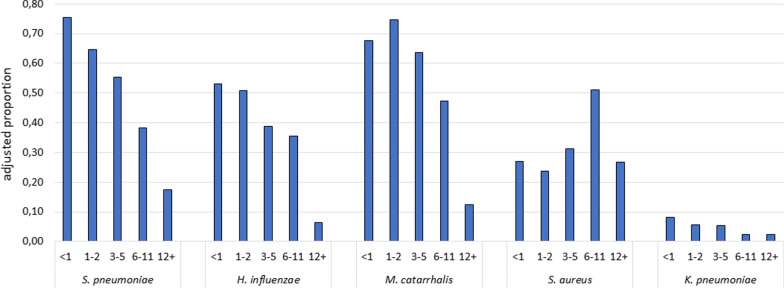
Prevalence of colonisation in different age groups. Prevalence estimated from a logistic regression model adjusted for by sex (male) and month of inclusion (January)

**Fig. 2:**
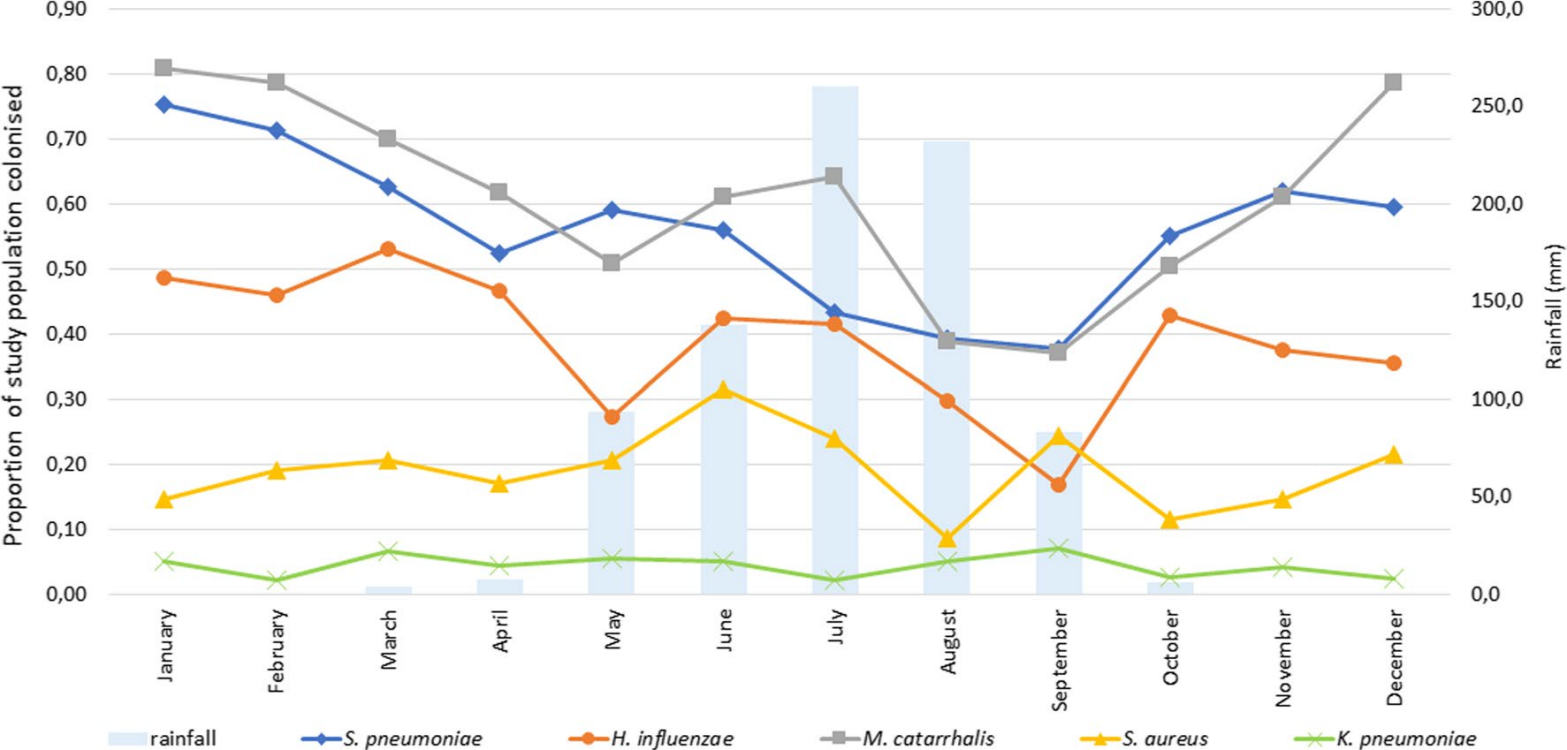
Proportion of study population colonized and rainfall during the study period by month of year. Proportions estimated from a logistic regression model adjusted for by sex (male) and age (5.0 years)

Overall, the unadjusted prevalence of carriage in our study group was 0.44 (*S. pneumoniae*), 0.30 (*H. influenzae*), 0.47 (*M. catarrhalis*), 0.17 (*S. aureus*) and 0.04 (*K. pneumoniae*). *K. pneumoniae* carriage was consistently below 10% in all age groups and throughout the year (Figs. 1 and 2). After adjustment for age and month of inclusion, females were overall less likely to be colonised with *S. pneumoniae* (OR 0.71, p = 0.022, 95% CI 0.53–0.95) and *M. catarrhalis* (OR 0.73, p = 0.044, 95% CI 0.54–0.99) than males.

The prevalence of colonisation with *S. pneumoniae*, *H. influenzae* and *M. catarrhalis* was highest in the age groups < 1 year and 1–2 years of age and then decreased sharply with increasing age. *S. aureus* colonisation showed a peak in the 6–11 years age group but was otherwise constant. *K. pneumoniae* carriage did appear to be higher in the younger age groups, although no statistically significant linear correlation was found (Fig. 1).

The colonisation rates for *S. pneumoniae*, *H. influenzae* and *M. catarrhalis* showed a marked seasonal association, with rates 2.0–2.9 times higher in January (dry season) as compared to September (rainy season). *S. aureus* colonisation over the year was more erratic and did not follow a clear seasonal pattern. *K. pneumoniae* colonisation rates did not significantly vary over the months (Fig. 2).

### Correlation between colonisation and clinical disease

**Fig. 3:**
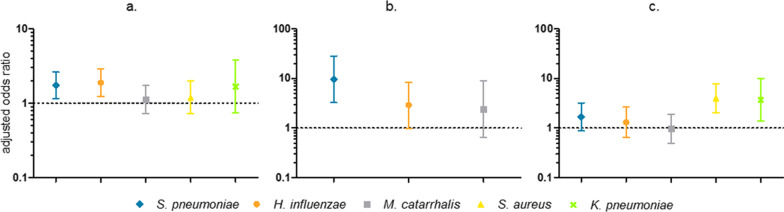
Adjusted odds ratio with 95% confidence interval for **a** discharge diagnosis of pneumonia in *S. pneumoniae*, *H. influenzae*, *M. catarrhalis*, *S. aureus* and *K. pneumoniae* carriers versus noncarriers, **b** bacteraemia with *S. pneumoniae*, *H. influenzae* and *S. aureus* in carriers of the same bacterium versus noncarriers, **c** mortality in *S. pneumoniae*, *H. influenzae*, *M. catarrhalis*, *S. aureus* and *K. pneumoniae* carriers versus noncarriers. Figures show the correlation between determinants (*S. pneumoniae*, *H. influenzae*, *M. catarrhalis*, *S. aureus* and *K. pneumoniae* carriage) and outcome variables [discharge diagnosis of pneumonia (**a**), bacteraemia (**b**) and mortality (**c**)], after adjustment for age, sex and month of inclusion, calculated using separate logistic regression models

Out of 924 patients who provided a nasopharyngeal swab, 155 were diagnosed with pneumonia. A total of 584 patients were diagnosed with one or more conditions other than pneumonia and the remaining 186 patients were discharged from the hospital with a diagnosis that neither in- or excluded pneumonia (e.g. ‘septicaemia’ without specification of the primary focus). We found higher odds for pneumonia in carriers of *S. pneumoniae* (OR 1.75, p = 0.008, 95% CI 1.16–2.63) and *H. influenzae* (OR 1.90, p = 0.004, 95% CI 1.23–2.92), but not *M. catarrhalis* (OR 1.13, p = 0.59, 95% CI 0.73–1.73) (Fig. 3a).

‘Bacteraemia’ was defined as the presence of a pathogenic bacterium either in blood culture or in blood qPCR. 88 patients had positive blood cultures, a further 40 patients had positive blood qPCR. In total, 128 patients (13.9%) were bacteraemic: 27 with *S. pneumoniae*, 20 with *H. influenzae*, 11 with *S. aureus*, 1 with *K. pneumoniae* and 72 with other bacteria (Additional file 1: Sheet 3). Three patients were bacteraemic with two different bacteria.

Colonisation with *S. pneumoniae* gave higher odds for *S. pneumoniae* bacteraemia (OR 9.63, p < 0.001, 95% CI 3.28–28.24). Nasopharyngeal colonisation, however, was not a prerequisite for bacteraemia: nasopharyngeal swabs for 5 out of 27 (18.5%) patients with pneumococcal bacteraemia tested negative for *S. pneumoniae*. In *H. influenzae* and *S. aureus*, the correlation between colonisation and bacteraemia was not statistically significant (Fig. 3b).

Remarkably, nasopharyngeal carriage of *S. aureus* was associated with bacteraemia and mortality, independent of disease caused by *S. aureus* itself. *S. aureus* carriers had similarly high odds ratios for bacteraemia with *S. aureus* (n = 11) (OR 2.44, p = 0.18, 95% CI 0.66–9.10, adjusted for age, sex and month of entry using a logistic regression model) and bacteraemia with other bacteria (n = 117) (OR 2.27, p = 0.001, 95% CI 1.42–3.63, adjusted for age, sex and month of entry using a logistic regression model) as compared to *S. aureus* noncarriers. Additionally, *S. aureus* colonisation was associated with lethal outcome (OR 4.01, p < 0.001, 95% CI 2.06–7.83) (Fig. 3c), even after adjustment for bacteraemia (OR 3.54, p < 0.001, 95% CI 1.79–7.02). We did not find this association in *S. pneumoniae*, *H. influenzae* or *S. aureus*. *K. pneumoniae* colonisation did appear to be correlated with mortality (OR 3.73, p = 0.009, 95% CI 1.38–10.07, adjusted for age, sex and month of entry using a logistic regression model), but with a very wide confidence interval due to low numbers.

### Influence of HIV, tuberculosis and malaria

**Fig. 4:**
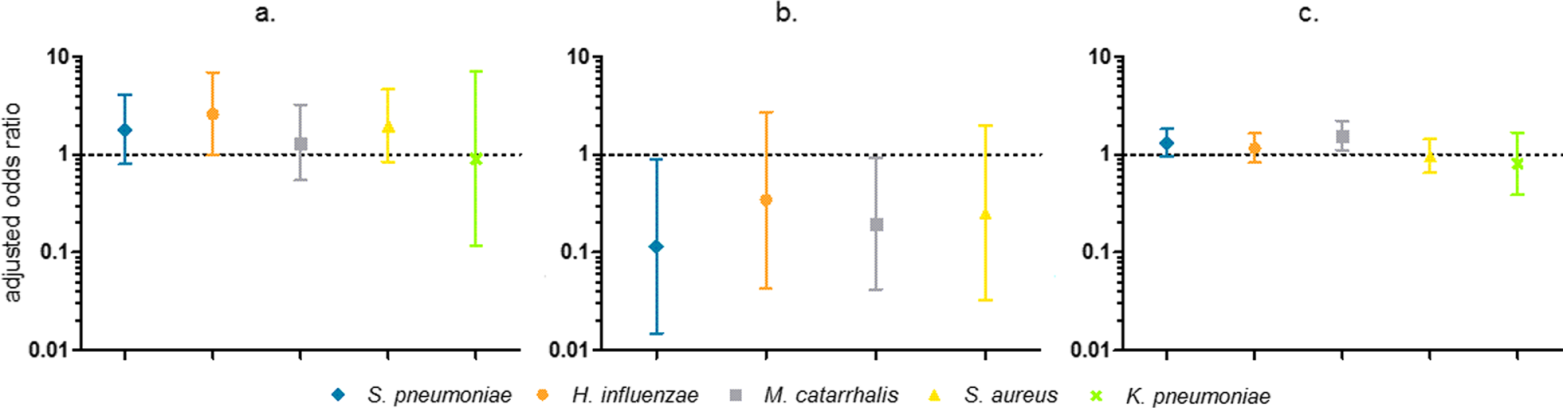
Adjusted odds ratio with 95% confidence interval for nasopharyngeal colonisation with *S. pneumoniae*, *H. influenzae*, *M. catarrhalis*, *S. aureus* and *K. pneumoniae* in **a** HIV positive versus HIV negative patients, **b** patients with versus patients without tuberculosis, **c** patients with versus patients without malaria. Figures show the correlation between determinants [HIV positivity (**a**), tuberculosis (**b**), and malaria (**c**)] and outcome variables (*S. pneumoniae*, *H. influenzae*, *M. catarrhalis*, *S. aureus* and *K. pneumoniae* carriage), after adjustment for age, sex and month of inclusion, calculated using separate logistic regression models

HIV positive patients (n = 33) had somewhat higher odds for colonisation with *S. pneumoniae*, *H. influenzae*, *M. catarrhalis* and *S. aureus*, although none of these correlations reached statistical significance (Fig. 4a). Patients diagnosed with tuberculosis (n = 20), on the other hand, were less likely to be colonised with *S. pneumoniae* and *M. catarrhalis* (Fig. 4b). No patients with tuberculosis tested positive for *K. pneumoniae*. The impact of malaria (n = 343) on nasopharyngeal colonisation was less pronounced (Fig. 4c), although a positive correlation with *M. catarrhalis* colonisation did reach statistical significance (OR 1.58, p = 0.01, 95% CI 1.12–2.22).

### Co-occurrence of carriage

**Fig. 5:**
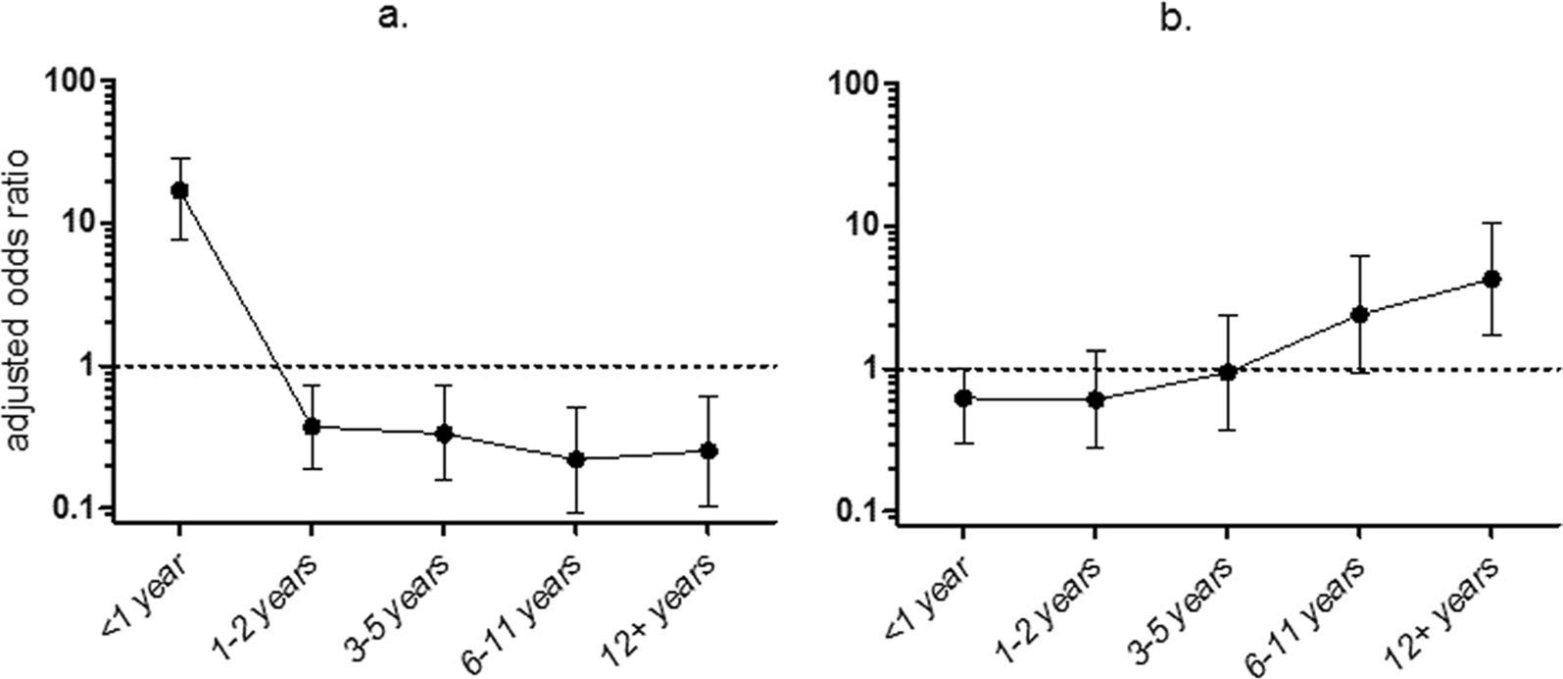
Effect of colonisation with **a** *M. catarrhalis* on colonisation with *S. pneumoniae*, **b** *S. pneumoniae* on colonisation with *S. aureus*. **a** Shows the correlation between the determinant (colonisation with *M. catarrhalis*) and outcome variable (colonisation with *S. pneumoniae*), after adjustment for age, sex and month of inclusion, calculated using a logistic regression model. **b** Shows the correlation between the determinant (colonisation with *S. pneumoniae*) and outcome variable (colonisation with *S. aureus*), after adjustment for age, sex and month of inclusion, calculated using a logistic regression model

643 patients (69.6%) tested positive for colonisation with at least one of the included bacteria. 416 of them (45.0% of the total) were colonised with multiple bacteria. The influence of nasopharyngeal colonisation with any of the five tested bacteria on colonisation with any of the four others was highly age-specific. In general, associations between carriage with *S. pneumoniae*, *H. influenzae* and *M. catarrhalis* were positive for children < 1 year and indifferent or negative in higher age groups, like for example the association between carriage of *M. catarrhalis* and *S. pneumoniae* (Fig. 5a and Additional file 1: Sheet 4). The odds *for S. aureus* colonisation generally rose for patients aged 12 years and older when colonised with *S. pneumoniae*, *H. influenzae* or *M. catarrhalis*, but in the younger age groups the opposite effect was visible (Fig. 5b and Additional file 1: Sheet 4).

## Discussion

In this study, we report a high prevalence of nasopharyngeal colonisation with *S. pneumoniae*, *H. influenzae*, *M. catarrhalis* and *S. aureus* in children and adults presenting to the hospital with an acute febrile illness in rural Burkina Faso. The prevalence of colonisation with *S. pneumoniae*, *H. influenzae* and *M. catarrhalis* was dependent on age and season. *K. pneumoniae* prevalence was low and not significantly correlated with age, season or carriage of other bacteria. The results suggest that nasopharyngeal carriage of *S. pneumoniae*, *H. influenzae* and *S. aureus* may be a risk factor for disease, supporting the clinical relevance of these epidemiological findings. *S. aureus* carriage was correlated with patient death independent of *S. aureus* bacteraemia. Additionally, carriage of some bacteria appeared to influence the carriage of others.

A major strength of this study is the high number of patients enrolled throughout the year, which allowed us to detect seasonal patterns and to correct for season in other analyses. Interestingly, colonisation rates were lowest towards the end of the rainy season. This may be due to ecological or climatic effects or, more likely, it could be an effect of anthropological or biological differences between seasons such as the effect of dust and dry air on the nasopharynx, crowding or seasonal effects on immune status [28]. Malaria did not appear to have a significant impact. Due to the setup of this study, which was originally designed to test and optimise an algorithm to distinguish malaria from bacterial infections in febrile patients, we were able to perform statistical analyses for a broad range of clinical parameters. The use of qPCR rather than culture allowed for detection of low grades of colonisation.

The most important weakness of this study is the inclusion of patients with a clinical suspicion of infection only. Because a significant proportion of the study population (16.8%) was diagnosed with pneumonia and all included pathogens are capable of causing pneumonia, the carriage prevalence in the general population is probably somewhat lower than the prevalence we presented in Figs. 1 and 2, especially for *S. pneumoniae*. A second limitation is the change in inclusion criteria: halfway through the study, non-hospitalised patients were also eligible for inclusion, meaning that on average, the study population would be less severely ill. Thirdly, it may be possible that fever itself has an effect on the nasopharyngeal microbiome. A final factor that may have influenced the results was the use of nasopharyngeal swabs only, as the combination of nasopharyngeal and oropharyngeal swabbing has been shown to yield higher carriage rates [19, 29].

Despite the inclusion of patients with a clinical suspicion of fever only, the nasopharyngeal carriage prevalences we found was comparable to other PCR-based studies in West Africa. Baggett et al. [15] reported a pneumococcal carriage prevalence of 88.6% in combined nasopharyngeal and oropharyngeal swabs taken from control subjects aged 1 month to 5 years in the Gambia, and 79.1% in Mali. Park et al. [20] reported carriage rates of 51.5% for *H. influenzae*, 74.3% for *M. catarrhalis* and 24.5% for *S. aureus* in children up to 5 years of age from 7 low- and middle-income countries, including the Gambia and Mali. Kwambana et al. [30] collected nasopharyngeal swabs from Gambian children repeatedly during the first year of life: 78% of swabs tested positive for *S. pneumoniae*. They also tested for the presence of *H. influenzae* (70%), *M. catarrhalis* (71%) and *S. aureus* (20%). It is impossible to directly compare these unadjusted prevalence data to ours, as prevalence is highly dependent on the parameters we adjusted for (age, sex and season). However, all reported prevalence falls within the range of the seasonal variation we found for the included age groups, with the exception of the pneumococcal carriage rate of 88.6% Baggett et al. reported for the Gambia, which was higher than the upper limit of the seasonal variation within children aged < 5 years in our study (52.9–86.0%).

We included *K. pneumoniae* in our study because it is known to be an important cause of CAP in low and middle income countries, comparable to *S. pneumoniae* in terms of frequency [5–7]. Carriage rates for *K. pneumoniae* were quite low in our population, however, especially when compared to *S. pneumoniae*. We also did not find the previously described pattern of higher prevalence of *K. pneumoniae* carriage among adults [5].

We found higher odds for pneumonia in carriers of *S. pneumoniae* (OR 1.75) and *H. influenzae* (OR 1.90). This correlation has previously been described for *S. pneumoniae*, *H. influenzae* and *M. catarrhalis* in other populations [15, 31] and may suggest that nasopharyngeal carriage of these respiratory pathogens is a first step towards invasive disease. Conversely, we did not find higher odds for pneumonia in *M. catarrhalis* carriers. Possible explanations for this contradictory finding are a lower pathogenicity of locally circulating *M. catarrhalis* or lower susceptibility to *M. catarrhalis* pneumonia in our population. As no sputum samples had been collected, we could not calculate odds ratios for nasopharyngeal colonisation with the pneumonia causing pathogen.

Nasopharyngeal carriage of *S. aureus* was associated with bacteraemia with non-*S. aureus* bacteria and, independent of this association, with 3.5 times higher odds for fatal illness. This could indicate that *S. aureus* carriers are at risk for more severe disease, potentially due to an immune-mediated mechanism. An alternative explanation would be that the immune response associated with severe, acute and potentially fatal infectious disease predisposes towards *S. aureus* colonisation. To our knowledge, this association has not been described before.

## Conclusions

Our study provides important insights into the dynamics of nasopharyngeal colonisation with bacterial pathogens during the rainy and dry season in rural Burkina Faso, the influence of age and sex and the association between carriage of different pathogens. The presented results may help understand the spread of these pathogens and could help to determine the cause of infection in febrile patients in these regions.

## Supplementary Information

**Additional file 1:** Additional tables.

**Additional file 2:** Overview of qPCR results.

**Additional file 3:** SPSS output file.

## Data Availability

The Additional file 1: Tables as well as the dataset containing the results of the PCRs performed on all samples, positive and negative controls and the SPSS output file are publicly available via https://osf.io/5c69q/.

## Abbreviations

CAP: Community acquired pneumonia
CMA: Centre Medical avec Antenne Chirurgicale
*H. influenzae*: *Haemophilus influenzae*
HIV: Human immunodeficiency virus
IRSS: Institut de Recherche en Sciences de la Santé/Clinical Research Unit of Nanoro
*K. pneumoniae*: *Klebsiella pneumoniae*
*M. catarrhalis*: *Moraxella catarrhalis*
PaluBac: Optimizing Sysmex technology as an innovative tool to differentiate between malaria (PALUdism) and BACterial infections in a malaria endemic region
qPCR: Quantitative polymerase chain reaction
*S. aureus*: *Staphylococcus aureus*
*S. pneumoniae*: *Streptococcus pneumoniae*
